# Genetic Approaches to Enhance Multiple Stress Tolerance in Maize

**DOI:** 10.3390/genes12111760

**Published:** 2021-11-04

**Authors:** Nenad Malenica, Jasenka Antunović Dunić, Lovro Vukadinović, Vera Cesar, Domagoj Šimić

**Affiliations:** 1 Division of Molecular Biology, Faculty of Science, University of Zagreb, Horvatovac 102a, 10000 Zagreb, Croatia; malenica@biol.pmf.hr; 2 Department of Biology, Josip Juraj Strossmayer University, Cara Hadrijana 8/A, 31000 Osijek, Croatia; jantunovic@biologija.unios.hr (J.A.D.); vera.cesar@biologija.unios.hr (V.C.); 3 Agricultural Institute Osijek, Južno Predgrađe 17, 31000 Osijek, Croatia; lovro.vukadinovic@poljinos.hr; 4 Faculty of Dental Medicine and Health, Josip Juraj Strossmayer University of Osijek, Crkvena 21, 31000 Osijek, Croatia; 5 Centre of Excellence for Biodiversity and Molecular Plant Breeding (CroP-BioDiv), Svetošimunska 25, 10000 Zagreb, Croatia

**Keywords:** maize, multiple-stress tolerance, quantitative genetics, genetic engineering

## Abstract

The multiple-stress effects on plant physiology and gene expression are being intensively studied lately, primarily in model plants such as Arabidopsis, where the effects of six stressors have simultaneously been documented. In maize, double and triple stress responses are obtaining more attention, such as simultaneous drought and heat or heavy metal exposure, or drought in combination with insect and fungal infestation. To keep up with these challenges, maize natural variation and genetic engineering are exploited. On one hand, quantitative trait loci (QTL) associated with multiple-stress tolerance are being identified by molecular breeding and genome-wide association studies (GWAS), which then could be utilized for future breeding programs of more resilient maize varieties. On the other hand, transgenic approaches in maize have already resulted in the creation of many commercial double or triple stress resistant varieties, predominantly weed-tolerant/insect-resistant and, additionally, also drought-resistant varieties. It is expected that first generation gene-editing techniques, as well as recently developed base and prime editing applications, in combination with the routine haploid induction in maize, will pave the way to pyramiding more stress tolerant alleles in elite lines/varieties on time.

## 1. Introduction

Maize (*Zea Mays* L.) is, together with wheat and rice, one of the three cereals that feed the world. As a major crop worldwide, maize is essential for industry and it is cultivated mostly in rainfed cropping systems where changes in climate, erosion and a dwindling water supply threaten to diminish future yields [1]. The most important trait in maize is grain yield, a composite trait that is influenced by many stress-related traits. Research on yield potential and stress tolerance in maize, combined with extensive phenotypic selection, has helped achieve significant genetic gains in rain-fed yield of maize hybrids [2]. Tollenaar and Lee [3] stated that the genetic yield improvement of North American maize varieties during the 20th century is closely associated with enhanced stress tolerance.

The main stresses that affect maize plant in the field are being extensively studied. They include drought [4,5], heat [6], chilling [7,8,9], flood [10], fungi and viruses [11,12], parasitic plants [13], insects [14], pesticides [15], herbicides [16,17], heavy metals [18,19], poor nutrients [20,21], soil salinity [22] and soil acidity [23,24]. Ongoing climate change is expected to aggravate this burden. Globally, it was projected that climate change could affect yield reduction in maize by an average of 7.4% for every 1 °C increase in mean temperature [25]. Yet, percentage yield change as a function of temperature is not the same in tropical and temperate regions [26] and it could even be positive according to the Intergovernmental Panel on Climate Change Fourth Assessment Report (IPCC AR4) [27]. However, if additional stress factors, such as nitrogen deficiency, are to be included, according to the results of seven global gridded crop models, a negative percentage yield change could be drastic, especially in tropical regions [28].

Drought, as the second most important cause of yield loss for maize after poor soil fertility, affects 20–25% of the global maize area each year [29]. Maize is quite drought susceptible compared to other cereals, with the exception of rice. This has considerable consequences, as most of the maize producing areas are under rainfed conditions [30]. Moreover, if a long-term drought occurs in either a jointing or tasseling period for more than 30 days, no plant recovery is possible, even after irrigation [31]. In order to escape drought, the coordination of phenology with water availability can be performed choosing maize genotypes of early maturity [32]. However, choosing early maize hybrids may not be an optimum option, since these hybrids can be more sensitive to heat stress than late hybrids [33]. Generally, the strategies of drought escaping or avoiding are commonly used in maize where stress can be circumvented by earlier planting dates or planting earlier hybrids to avoid the assumed adverse weather conditions mostly during flowering. However, the global trends in temperature and precipitation suggest that extreme weather events may occur at any time throughout the growing season, including a cold spring and late spring frost, thus making an early planting date or early genotypes not worthwhile [33]. Thus, it seems that seeking for drought resistance is actually seeking for drought tolerance, defined as a potential for plants to maintain their growth and development under drought stress [32]. Aslam et al. [32] gave an overview of numerous adaptation mechanisms at the physiological and molecular levels conferring drought tolerance. At the physiological level, they include osmotic adjustment, antioxidative defense mechanism and plant growth regulators. Molecular mechanisms comprise stress proteins and water channel proteins, transcription factors and signal transduction pathways.

Up until recently, research on heat stress in maize is not as exhaustive as research on drought. However, Lobell et al. [34] demonstrated that extreme heat as a stressor had a more critical role for maize production than drought in the US, corroborating previous statistical studies of rainfed maize yields showing a strong negative yield response to the accumulation of extreme temperatures (>30 °C) and a relative weak response to seasonal rainfall. A leaf temperature above 30 °C affects net photosynthesis because of rubisco inactivation [35], but there is notable acclimation when the temperature increase was gradual (2.5 °C h^−1^ from 28 to 45 °C) rather than abrupt (1 °C·min^−1^ for the same temperature range) [36]. Hasanuzzaman et al. [37] listed two major effects of heat stress in maize during flowering on plant and ear growth rates, and in the reproductive stage on ear expansion. Similar to drought, there are several adaptation mechanisms at the physiological and molecular levels conferring heat tolerance, such as the following: osmoprotectants, antioxidative defense, expression of stress proteins, signaling cascades and transcriptional control [37].

Another abiotic, e.g., an anthropogenic stressor important for crop production, is heavy metal excess in the soil, representing a threat on the environment due to its toxicity to plants, animals and humans. The most important heavy metal in maize is cadmium that induces growth inhibition, changes in the water and ion metabolism, the inhibition of photosynthesis, changes in enzyme activities and the formation of free radicals [18,38]. Defense mechanisms comprise immobilization, synthesis of phytochelatins, as well as similar physiological mechanisms present under drought and heat conditions, including an accumulation of stress proteins, proline and salicylic acid [18]. Generally, responses to heavy metal toxicity involve an accumulation of reactive oxygen species (ROS), abscisic acid (ABA) and stomatal closure, but an antioxidant defense can be distinct in different organs when maize plants are subjected to sub-lethal concentrations of cadmium, copper, nickel and zinc [39]. Further, physiological implications and the toxicity of chromium, copper and mercury in the maize plant were also assessed [40]. A special case of an anthropogenic stressor in maize is high plant density. It is associated with nitrogen use stress and drought [41] due to plant competition generating multiple-stress environments and eventually affecting plant physiology, phenology and morphology [42].

Beside European Corn Borer (*Ostrinia nubilalis* L.), the two most prominent biotic stressors in maize are Diabrotica and Fusarium, which are interrelated with abiotic stressors [43,44]. The western corn rootworm (*Diabrotica virgifera virgifera* LeConte) is one of the major pests of maize in Europe and in the USA. Once detected, it is very difficult to eradicate as well as to manage. Efforts for identifying sources of resistance to corn rootworm within maize cultivars are crucial [45]. Apart from studying natural genetic variation via quantitative genetics and classical breeding, several biotech companies developed the first transgenic (genetically modified-GM) maize cultivars by transferring insecticidal protein gene(s) from bacterium *Bacillus thuringiensis*, Berliner (*Bt*) into maize (‘Bt maize’) [46], as an alternative to chemical control and crop rotation for insect control. However, the development of maize hybrids with native resistance to insects would be a sustainable management tool [45].

Climate change resulting in higher temperatures and less rainfall, largely favors the development of fungus *Fusarium sp*. *Fusarium verticillioides* is less pathogenic and a higher disease intensity only occurs if the plant has been previously weakened by other biotic or abiotic stress [47]. The infection of maize with *F. verticillioides* can lead to a contamination of the grain with mycotoxin fumonisin synthesized by fungi during development. Campos-Bermudez et al. [48] presented a combination of biochemical and molecular approaches to clarify metabolic changes following maize infection with *F. verticillioides*.

**Figure 1:**
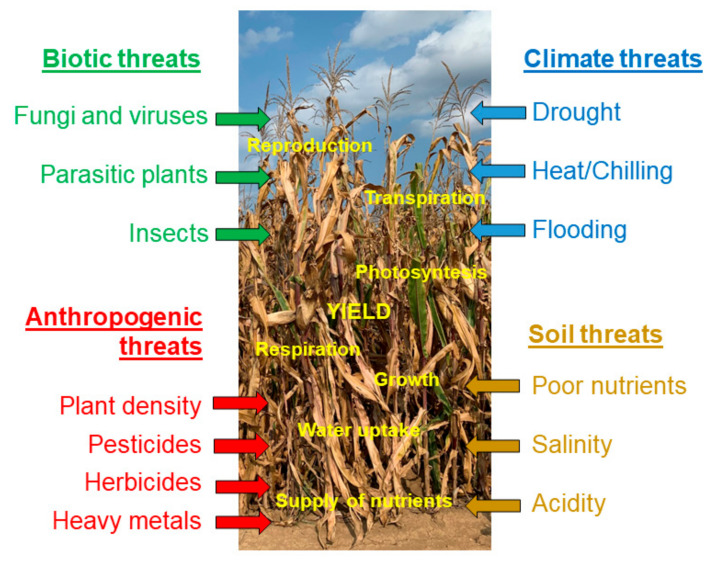
Most important stress factors in maize. Adapted from Zandalinas et al. [53].

Due to the complex interaction among different stresses, only a tiny fraction of studies on plant responses to stresses deals with the combinations of two or more stresses. A closer inspection of the database search revealed that only about 1% of the original articles had stress combination as a subject [49]. However, this could be changed due to extensive research on climate change and its multifactorial nature affecting unpredictable combinations of different stresses and posing an even greater threat to major crops [50]. Mittler [51] proposed a stress matrix approach to visualize the individual positive and/or negative interactions among different stresses and their overall effect on plant growth and yield. At this two-dimensional level, the majority of the interactions among the stresses seem to be negative [52]. When more than two stress factors co-occur, a severe decline in the subsequent plant growth and survival takes place. Zandalinas et al. [53] defined a multifactorial stress combination as a combination of three or more simultaneous stress factors grouping in the following four major threats: anthropogenic, biotic, climate and soil threats. The four threats that include agronomically important stress factors in maize are presented in Figure 1. They affect the supply of nutrients, water uptake, growth, respiration, photosynthesis, transpiration, reproduction of maize plant and, eventually, yield. Once additional stresses are presented, even at low levels, they could negatively interact with each other and bring about substantial decreases in agricultural productivity [53].

The most investigated stress combination in maize is drought and heat, predominantly in African environments [54,55,56,57,58,59]. In a simulation study, it was demonstrated that incorporating combined drought and heat tolerance into maize tropical varieties may increase grain yield under both the baseline and future climate scenarios [60]. In Africa, there is also a research interest in multiple-stress tolerance to drought and the parasitic plant of *Striga hermonthica* [61], as well as to a *Striga*, low soil nitrogen and drought combination [62]. In tropical rainfed environments in general, climate-induced stressors are drought, heat, waterlogging, salinity, cold, diseases and insects, which frequently come in different combinations [63]. Elsewhere, there are studies on the interactions between insects and other stress factors such as salinity [64], flooding [65] and on the interaction between pesticides and salinity [66]. The combined stress of flooding and infestation with the insect pest *Spodoptera frugiperda* (fall armyworm) leads to an elevated production of salicylic acid, which does not occur in the individual stresses [65]. Studies on a combination of the three simultaneous stress factors in maize are scarce. The combination of *Striga*, low soil nitrogen and drought was investigated in Nigeria [62]. Based on the multiple-trait selection index, the top low-N, *Striga* and drought tolerant/resistant maize genotypes were recognized as vital sources of beneficial alleles for the improvement of tropical yellow maize germplasm. The combination of drought, insect infestation and Fusarium in the context of fumonisin contamination was examined in California [67]. The significant main effects of hybrid, planting date, insecticide treatment and drought stress on Fusarium ear rot symptoms and fumonisin B1 contamination were detected, and these factors also had significant interacting effects. Recently, the combination of drought, heat and the effect on some arthropod pests was reviewed [68] focusing on mechanisms of physiological, biochemical and molecular responses previously given by Aslam et al. [32]. To conclude, Chávez-Arias et al. [68] summarized the following impacts of the combination of heat and drought on the physiological responses of maize plants: decrease in yield, increase in days from anthesis to silking, reduction in growth parameters (height, stem diameter, leaf area, fresh and dry weight), changing water status and nutrient content, decrease in chlorophyll content and gas exchange parameters such as photosynthesis, stomatal conductance and transpiration, reduction in fresh and dry weights, as well as increase in leaf temperature.

## 2. Physiological Traits for Screening Genotypes for Multiple-Stress Tolerance

While many stress responses appear to be stress-type-specific, it is clear that some stress responses are more general and potentially confer tolerance to multiple types of stressors [69]. As mentioned above, an accumulation of some osmoprotectants, such as proline and other amino acids, or changing the concentrations of some antioxidants represent a plant response on several individual stressors (e.g., drought, heat, heavy metal toxicity). By investigating leaf metabolites and yield under drought, heat and combined drought and heat conditions in the field, Obata et al. [70] found that drought stress evoked the accumulation of various amino acids (isoleucine, valine, threonine, 4-aminobutanoate, glycine, serine and myoinositol), while the combination of drought and heat evoked relatively few specific responses. Most of the metabolic changes were predictable from the sum of the responses to individual stresses. However, drought stress can have a predominant effect overheat stress, although total soluble sugars, proline and total free amino acids were increased under all the stress treatments [71].

Abiotic stress factors produce highly reactive forms of oxygen capable of rapidly reacting with and oxidizing numerous cellular constituents (e.g., proteins, lipids, DNA, RNA) depending on stress severity and duration. These toxic intermediates, reactive oxygen species (ROS), can disturb the metabolic processes of cells and, consequently, lead to cell death. Plants have evolved efficient antioxidant mechanisms, enzymatic and non-enzymatic, to cope with ROS overproduction. A large number of ROS detoxifying proteins (superoxide dismutase (SOD), catalase (CAT), ascorbate peroxidase (APX), guaiacol peroxidase (POD), glutathione peroxidase (GPX), glutathione reductase (GR), etc.) and non-enzymatic antioxidants such as ascorbic acid or glutathione are present in cells. The details of ROS production and ROS defense pathway were documented and discussed in numerous review papers [72,73,74,75,76]. The substantial imbalance between ROS production and scavenging has been shown in maize and other crops dealing with cadmium toxicity [77,78] or drought [79,80,81,82]. However, the antioxidative response proved to be strongly genotype-dependent. Oxidative damage and the antioxidative response were different in the leaves of two diverse maize inbred lines [83,84] subjected to excess cadmium in soil, water limitation as well as to a combination of both stress factors [85]. A gradual significant increase in POD activity was observed in one inbred line across the following three treatments: Cd excess, water limitation and a combination of both, but not in other one. The opposite case was with proline activity where a dramatic increase occurred only in the second inbred line under water limitation and stress combination conditions.

The protective role of proline in osmotic adjustment is well documented [86,87,88]. Increased proline indicated that cadmium provoked certain genotypes to synthesize more proline to resist osmotic stress, as also assumed by Zhao et al. [89].

These results suggest that different strategies of antioxidative mechanisms could be present in maize inbred lines subjected to water limitation, excess cadmium and a combination of both stress factors, indicating existing multiple-stress tolerance in some genotypes. The same is true for the combination of drought and heat [54] to identify combined drought and heat tolerant donors. However, there was a genotype by trial interaction, and tolerance to combined drought and heat stress was genetically distinct from tolerance to individual stresses, whereby tolerance to either stress alone did not confer tolerance to its combination. Nevertheless, tolerance to drought, heat, cadmium toxicity and other stressors under multiple stress conditions requires further extensive genetic and physiological research in order to identify and use tolerant genotypes for breeding for an adaptation under climate change.

All stress factors, directly or indirectly, inhibit the most important physiological process of photosynthesis as a global sensor of environmental stress in plants [90]. In the last few decades, chlorophyll *a* fluorescence measurement, as a rapid and non-destructive method, was recognized as a useful tool for screening plant sensitivity to various stress factors [77,78,79,91,92]. The illumination of dark-adapted leaves using actinic light for 1 s enables a polyphasic chlorophyll *a* fluorescence induction curve to be obtained (O-J-I-P transient). An interpretation of the JIP-test, a mathematical model developed by Strasser [93], provides exhaustive information dealing with the structure and function of the photosynthetic apparatus [91,93].

Numerous studies have shown different responses provoked by adverse environmental features, depending on the crop species, genotype, stress type and duration and developmental stage. The fluorescence parameters are being used extensively in stress physiology in a range of plant species under controlled conditions and it is also easily adaptable to field conditions [94]. This is particularly important for maize, because stress studies conducted under controlled conditions inadequately reflect natural environmental conditions. While the impact of the following individual stress factors on maize’s photosynthetic efficiency has been well documented: drought [94,95,96], heat [35,97], chilling [98], fungi [99], herbicides [100], herbicides and cadmium [101], cadmium alone [84,102], low nitrogen [103], soil salinity [64,104,105] and plant density [106], there is a lack of information dealing with the multiple stress tolerance. Qu et al. [107] have monitored the effect of a combination of potassium deficiency and salt stress in maize seedlings grown in controlled conditions. The obtained results revealed that a combination of investigated stress factors impaired the light reaction pathways of PSI and PSII and resulted in severe photochemical damage in leaves if compared with the ones affected by individual stress factors. Correia et al. [108] investigated the contrasting levels of tolerance to drought and heat of two maize genotypes, B73 (heat and sensitive) and P0023 (drought-tolerant hybrid). Limited transpiration under heat and drought allowed water savings to act as a drought stress avoidance mechanism. A higher phosphorylated phosphoenolpyruvate carboxylase (PEPC) and electron transport rate (ETR) in P0023 maintained the photosynthetic efficiency. A limited transpiration rate and a synchronized carbon assimilation regulation were identified as the key traits for drought and heat tolerance in maize [108]. A combination of excess cadmium and drought stress were tested in lines Os6-2 and B84, and in their hybrid [109]. A decreased total performance index, PI_tot_, a parameter that comprises the functional activity of photosystem II, photosystem I and intersystem electron transport chain, was reported in Os6-2 and in hybrid. The destabilization of the oxygen evolving center of PSII (OEC) and a lower PS stability were also observed. Photoinhibition due to the stress combination also occurred in line B84, despite an unchanged PI_tot_, the most sensitive parameter of the JIP-test. The results suggested that all the investigated maize genotypes have developed different strategies to cope with a combination of excess cadmium and drought. The possibility of obtaining exhaustive information about leaf photochemistry during real-time in situ monitoring in field conditions, as well as the other technical advantages of chlorophyll *a* fluorescence approaches, have made it a popular technique for crop phenotyping.

## 3. Strategies for Enhancing Multiple-Stress Tolerance in Maize

### 3.1. Natural Genetic Variation

The main genetic approach to the enhancement of multiple-stress tolerance is utilizing the natural genetic variation of plant quantitative traits associated with stress tolerance where quantitative genetics play a pivotal role via classical and molecular breeding. Although quantitative genetics has entered the second century, it still serves as the genetic basis of contemporary plant breeding [110,111] and maize breeding in particular [112]. Virtually all the studies on stress tolerance already mentioned in this review are directly or indirectly related to classical maize breeding. In this chapter, the focus is on molecular breeding, i.e., genetic mapping identifying quantitative trait loci (QTL) [113] and genome-wide association studies (GWAS) [114] on multiple-stress tolerance traits in maize. Briefly, maize genetics and a genomic database [115] search using the keyword “stress” within a “Loci+QTL” data subset [116] retrieved a total of 90 genes and loci directly related to stress factors. Among others, there are 39 genes belonging to *aasr* (Abscisic acid-ABA stress-ripening) and *hsftf* (heat stress transcription factor) families, as well as “universal/general stress protein” genes *pco103004*, *pco143261*, *pco144726* and *pco152469.* There are a total of 27 retrieved quantitative trait loci: 20 associated with drought (“qgyldws” loci), 6 “stressed-leaf ABA content” QTL (“qslaba” loci) and one “unstressed-leaf ABA content” QTL (“qulaba” locus). Otherwise, there is a plethora of QTL experiments dealing with stress in maize: according to Web of Science Core Collection Database [117], more than 400 articles are currently to be retrieved in this regard. On the other hand, the number of studies on molecular breeding for multiple-stress tolerance in maize is limited, although advanced models for multi-trait QTL analysis were known for more than a decade, (e.g., [118]).

In a field experiment including stress treatment blocks (drought, low nitrogen and combined), Makumburage and Stapleton (2011) [119] examined phenotype uniformity and mapped QTL in IBM94 intermated recombinant inbred lines [120]. They concluded that phenotype uniformity, which is genetically controlled, has a different genetic architecture in multiple-stress environments compared to single-stress blocks. In an associated article, the genetic architecture of drought and ultraviolet radiation stresses along with their combination was examined in two maize mapping populations, revealing the complex attenuating interactions among physiological signaling steps in two stress responses [121]. Recently, combined drought and heat stress tolerance was examined in the DTMA (Drought Tolerant Maize for Africa) association-mapping panel [122], including 300 tropical and subtropical maize inbred lines genotyped with genotyping-by-sequencing (GBS) [123]. This GWAS mapping revealed few overlapped significant markers and candidate genes for the same traits across different stress environments, showing the genetic divergence between the individual stress tolerance and the combined drought and heat tolerance. Another GWAS mapping revealed that only one GWAS-base candidate gene was associated with each of the five of the six combined insect resistance quantitative trait nucleotides (QTNs), thus supporting the pleiotropy hypothesis [124]. Nevertheless, the delivered multiple insect resistance physical map should contribute to the possible enhancement of combined insect resistance in maize. Hou et al. [125] used an IBM Syn10 DH population [126] to detect the quantitative trait loci (QTL) for a combined lead and cadmium tolerance by linkage mapping in maize seedlings contributing to functional gene identification and molecular breeding for improving heavy metal tolerance.

Quantitative genetic studies have identified genetic correlations among stress-resistance traits, such that the selection of tolerance to one type of stress has been associated with tolerance to another type of stress as a correlated selection response [127]. However, due to a significant genotype by environment interaction, these associations are generally not clear. Furthermore, genetic correlations for grain yield between the means under drought and combined drought and heat conditions, as well as under heat and combined drought and heat conditions were negligible (0.08 and −0.07, respectively) [54]. At the molecular level, certain heat-shock proteins are commonly elicited in response to various stress conditions [128]. The Putative pleiotropic candidate gene for the quantitative trait locus (QTL) detected on maize chromosome 7, associated with several chlorophyll fluorescence parameters, seemed to be *gst23* [94]. It belongs to the large glutathione transferase gene family that encodes glutathione transferase, which have an important role in plant responses to abiotic and biotic stresses [73]. Mullineaux and Karpinski [129] stated that in *Arabidopsis*, some compounds (oxylipins) during abiotic and biotic stresses may induce the expression of a *gst* gene. The pleiotropy of a *gst* gene was identified for three maize diseases [130], suggesting the importance of glutathione transferase in response of maize to biotic stress. However, Wallace et al. [112] stated that the majority of QTLs are not pleiotropic and the presumed correlation between quantitative traits seems to be due to the population structure. The putative association of the *gst23* gene with five chlorophyll fluorescence parameters might suggest that glutathione transferase is linked with the regulation of photon absorbance and exciton dissipation, as well as in the trapping/dissipation ratio and, therefore, provides biological and biochemical plausibility that this member of the maize *gst* family is associated with photosynthetic efficiency in general, and eventually with multiple-stress tolerance. The same might be true for maize Mitogen-Activated Protein Kinase Kinase Kinase (MAPKKK) [131]. In transgenic Arabidopsis, NDP kinase interacts with two MAPKs enhancing the multiple-stress tolerance [132].

Generally, stress tolerance is a polygenic trait controlled by many genes, and most of these genes have minor effects [133]. Therefore, classical linkage or QTL analysis in maize achieved limited success in improving polygenic complex traits due to the low power of detecting minor effects, coarse mapping and capturing limited genetic diversity in mostly biparental populations. However, some stress-related traits such as cadmium accumulation seem to be controlled by only a few major genes [83] reconsidering the classical marker assisted selection as a tool for contemporary plant breeding [134]. On the other hand, GWAS was more successful in the identification of thousands of genomic regions associated with many stressors, revealing a natural variation for stress tolerance and physiological traits in the diverse genetic material of maize [135]. GWAS has also some disadvantages though, including providing false positive/false negative associations and a weak identification of rare allelic variants [135]. Nevertheless, the ultimate dissection of a phenotype can only be completed with a direct connection with a DNA sequence variation [136], which the QTL/GWAS framework has not been able to provide thus far.

### 3.2. Genetic Engineering

In addition to traditional breeding and molecular breeding approaches, in the last two decades, we have witnessed a global expansion of genetically engineered (genetically modified—GM), i.e., transgenic, maize varieties. Since the approval of the first transgenic maize varieties such as MON 809 and MON810 in the USA in 1996, the numbers of authorized and cultivated GM maize varieties are steadily rising. Currently, three comprehensive databases of cultivated GM crops hold the following entries for GM maize: the *International Service for the Acquisition of Agri-biotech Applications* (ISAAA) lists 240 [137], the Biosafety Clearing House (BCH) 298 [138] and the *Food and Agriculture Organization GM Foods Platform* (FAO), 179 entries [139]. A recent extensive meta-analysis showed that GM maize varieties, during their two decades of cultivation (1996–2016), resulted in multiple benefits for the farmers, consumers and the environment by increasing the grain yield, reducing the mycotoxin content and sparing non-target organisms, respectively [140]. The same study reports that GM maize represents one-third of all the planted maize globally. In the context of multiple-stress tolerance, the first GM maize varieties, e.g., MON809, was already designed to fight the following two biotic stressors simultaneously: weeds, via its herbicide (glyphosate) tolerance trait [120], and lepidopteran insect resistance, via the encoded *Bt* toxin family protein, Cry1Ab endotoxin. MON809 was produced by biolistic cotransformation and the cointegration of a plasmid harboring the *Cry1Ab* gene and a second plasmid harboring *C4 EPSPS* and *gox* genes for glyphosate tolerance. However, in this particular case, the glyphosate tolerance trait was not efficient in field conditions. Therefore, the desired trait combination of insect resistance and herbicide tolerance was added by conventionally breeding two single-trait varieties, generating one of the first two-stacked events MON810 × MON88017 [120]. In principle, the term *stacking* means to combine several traits in a single maize variety. It can be achieved in the following ways: (a) by the cotransformation/cointegration of two or more T-DNA plasmids/linear DNA fragments [141] such as in the case MON809; (b) by transgene design, where all the traits/genes are cloned into one T-DNA or biolistic DNA fragment the like in the case of “Golden rice” harboring three genes on a single T-DNA [142]; (c) by conventional crossbreeding of different thoroughly characterized single-trait GM varieties, the so-called *events*. Stacking by crossbreeding is favored over the cotransformation or retransformation approaches [141] for regulatory reasons: national authorities tend to fast track breeding stacks generated by single-trait GM varieties that have been previously characterized and approved. On the other hand, the retransformation of an approved GM maize variety with another T-DNA construct would have to be evaluated de novo.

Additionally, a point to consider is that stacked events have, in principle, a hemizygous genotype, i.e., they have just one and not two T-DNA copy per locus. This is because commercial stacked events are F1 hybrids of elite inbred lines. As new traits are added, the generation of corresponding parental lines becomes more time-consuming. Fortunately, maize breeders have a genetic tool to make this problem easier to tackle: haploid inducer lines. The Stock6 haploid inducer was described decades ago [143] and was used commercially without the understanding of the molecular mechanism. Recently, it was shown that a mutation in *MATRILINEAL* (*MTL*), a pollen-specific phospholipase, is responsible for the HI phenotype of Stock6 [144,145]. In addition, another haploid inducing approach was established in maize, which is based on the previously CENH3-mediated haploid induction [146,147]. In maize, the haploid inducing capacity is provided by the heterozygous (+/*cenh3*) genotype. These HI lines will have an important role in increasing the breeding speed of maize multiple-stress tolerant stacks in the future.

Today, maize breeding stacked events are the most represented multi-stress tolerant commercial varieties. For example, 79% of the maize acres in the USA were planted with stacked varieties in 2020 [148]. Thus far, the following three stress-related traits have been successfully stacked in maize: insect resistance, herbicide tolerance and drought tolerance. Currently, the most complex maize stacks in cultivation are constituted of up to six different GM varieties (Table 1).

The six-stacked varieties were produced by the classical cross-pollination of six independent single-trait varieties, which were previously approved for cultivation [149]. However, the number of expression cassettes per variety is higher: some of the six-stacked varieties have up to three different herbicide resistance genes, up to six different natural or chimeric Cry proteins as well as one dsRNA expression cassette conferring insect resistance via the RNA interference pathway. Concerning possible interactions of individual transgenes in multi-stacked events, it is possible in theory, but highly unlikely, because the stacks were generated by the crosspollination of single events. Each single event was inserted in the maize genome randomly and independently. Therefore, the chance of inter-event recombination is not significant.

Moreover, stacking multiple insect resistance genes not only helps to improve yields due to warding off more insect types, but also due to the reduction in the mandatory non-GM refuge area proposed by the regulatory agencies, e.g., the Environmental Protection Agency (EPA). In the USA, a reduction in the non-*Bt* corn area from 20 to 5% [150,151] is allowed for stacked maize varieties.

The evolution of Bt resistance was slowed down and is still under control in countries that promote the mandatory planting of non-GM maize refuge areas. On the contrary, herbicide-resistant weeds appeared soon after the beginning of a mass application of glyphosate. There are the following strategies for controlling weed resistance: 1. increasing the herbicide concentration to the maximum a GM plant can tolerate; 2. increasing the copy number of herbicide-resistance genes by stacking (see Table 1); and 3. combining different herbicide resistance genes. Although immediately effective, none of these strategies is a sound long-term solution. In retrospect, it would have been reasonable that mandatory yearly crop rotation was promoted, or that a yearly exchange of different herbicides in combination with the corresponding resistance genes were applied.

The number of traits and genes that will need to be transformed or crossed into elite maize lines will grow in the future. Besides the current crossbreeding strategy, other advanced techniques for introducing large number of genes are in the pipeline, including the following: polycistronic expression in chloroplast [152], Binary Bacterial Artificial Chromosomes [153] or mini chromosomes [154,155]. However, all of these approaches still await routine use.

Apart from the classical transgenic approach, genome editing is the next breeding advancement that will enable new multiple-stress tolerant variety development [156,157]. The first use of the sequence-directed endonucleases such as TALEN and CRISPR/Cas9 in maize was demonstrated in protoplasts [158]. In addition, gene editing in planta was demonstrated on immature maize embryos, which were targeted by biolistic transformation. Maize monoallelic and biallelic mutations were successfully identified at *ASL2* (*acetolactate synthase*), *LIG1* (*liguleless1*) and two fertility loci, *Ms26* and *Ms45* [159]. Importantly, some edited plants were not transgenic, indicating only a transient expression of the Cas9-gRNA plasmid. This is important in respect to the negative public perception of transgenic plants. Svitashev et al. (2016) [160] further demonstrated the use of pre-assembled Cas9-RNA ribonucleoproteins (RNPs) for successfully mutating the same four loci and eliminating the possibility of binary vector genomic integration. In addition, RNP’s editing efficiency was approximately equal to the plasmid approach, whereas the off-target editing was not detectable. A more detailed study measuring off-target mutations in maize showed that the frequency of imprecise edits is negligible in comparison to the existing variation being naturally generated in the genome during the conventional breeding [161].

Another example was the editing of *ARGOS8*, a negative regulator of the ethylene response, which was sufficient to increase the maize grain yield under drought stress conditions [162]. By using a CRISPR/Cas9 homology-directed repair (HDR), the maize promoter *GOS2* was inserted downstream of the native *ARGOS8* promoter, leading to moderate levels of constitutive expression and leading to better performance under drought conditions. Genome editing also enables the easy manipulation of elite hybrid parental lines, bypassing backcross breeding during the introgression of recessive mutant alleles. In particular, a recent study demonstrated successful editing of the *waxy* (*Wx*) locus, leading to high endosperm amylopectin content, which is necessary for industrial starch production [163]. Another example is the inactivation of two aldehyde dehydrogenase genes in maize (*ZmBADH2a* and *ZmBADH2b*), leading to the creation of the world’s first aromatic maize [147]. Besides inducing a small modification at single loci, it was recently demonstrated that big chromosomal rearrangements in maize are also possible with the CRISPR/Cas9 system. A targeted 75.5-Mb pericentric inversion was performed on maize chromosome 2 in order to re-open previously inaccessible chromosome regions for recombination [164].

Base editing and prime editing [165,166] are two novel gene editing techniques that do not induce DNA double strand brakes (DSB) and INDELs (insertions/deletions). Both have been applied in maize recently. For example, maize protoplast and immature maize embryos were transformed with a cytidine deaminase attached to an inactive nCas9 variant targeting the *ZmCENH3* locus in order to obtain edits necessary to generate a haploid-inducing phenotype. Monoallelic or biallelic C to T conversions were reported in both protoplasts and immature embryos [167]. Another example was the generation of sulfonylurea-resistant maize plants using a CRISPR/Cas9 nickase-cytidine deaminase (CT-nCas9) fused to an uracil DNA glycosylase inhibitor (UGI). The nickase induces one DNA nick and deaminates C to U, whereas a UGI prevents the repair of the newly generated uracil. Two loci, *ZmALS1* (acetoacetate synthase) and *ZmALS2,* were successfully edited, though with somewhat lower efficiencies for *ZmALS2*. Nevertheless, sulfonylurea resistant plants were obtained and the transgene itself was crossed out. The edited plants could withstand sulfonylurea concentrations 5–15 times the recommended upper limit. Another group achieved double mutants in the same two *ALS* genes, but this time using prime editing and reported an improvement in the editing efficiencies compared to the published pioneering work in rice [168]. Recently, to test the precision of prime editing in plants, off-target modifications or genomic pegRNA integrations (prime editing gRNA) were measured. Such events were reported to be negligible or nonexistent [169].

Another important development in improving the breeding speed of multi-stress tolerant maize variants in the future will be the combination of the genome editing and haploid induction. In this approach, named HI-Edit (*haploid induction editing technology*) or IMGE (*Haploid-Inducer Mediated Genome Editing*), the haploid inducer line is also the editor line with an integrated Cas9-gRNA expression cassette. After the HI inducer cross, the desired genome edit takes place in the zygote, followed by a uniparental genome elimination (haploidization). The product is a genome edited haploid that can be converted to a double haploid (DH) after cytostatic, i.e., colchicine, application [170,171]. This strategy is faster at generating edits to elite breeding lines, circumventing selfing or backcrossing if necessary for eliminating the transgene encoding the editing machinery.

To conclude, the multiple-stress tolerant maize varieties already in use are designed to primarily be weed protected (herbicide tolerance) and insect resistant (*Bt* toxins and RNAi). Only a minority of these varieties are also drought resistant, but this trait is expected to be introduced more frequently in new elite varieties. Nevertheless, a lot of other stress types have to be addressed in order to make agriculture more resilient to the consequences of climate change. Molecular tools and breeding techniques for achieving this goal seem to be at hand. A far more unpredictable challenge in reaching these goals will be public mistrust and politics [172].

## 4. Conclusions and Prospects for the Future

Understanding the genetic mechanism on individual and multiple stress tolerance will be limited unless physiological and molecular mechanisms of plant responses to individual stresses and stress combinations, consisting of both shared and unique responses [173,174], can be elucidated. Thus, an integrated framework is needed for this challenging task. Bailey-Serres et al. (2019) [175] presented the integrative potential of plant sciences exploring emerging strategies for enhancing sustainable crop production that could be also applied on multiple-stress tolerance. In this paper, we focus on a quantitative genetic approach based on naturally occurring variation and genetic engineering, as a result of integrating the meta-analysis of existing plant omics data [49]. However, the opportunities for enhancing multiple-stress tolerance in crops are much broader, comprising an investigation of beneficial soil and leaf microbiome, small-molecule delivery and the use of sensors (cellular, organ canopy and remote) [175], indicating the complexity of the topic. Research in *Arabidopsis* shows that six stress combinations [176], but already two stress combinations [177], are lethal, even when each single stress is relatively mild. This makes multiple stress tolerance a rather difficult goal to achieve. Nevertheless, systemic ROS signaling was identified as the crucial switch for integrating responses to at least abiotic stress combinations. Altogether, modern research on the genetic improvement of multiple stress tolerance should be more connected to molecular phenotype modeling by using classical crop modeling [178] or machine learning approaches [179,180]. Machine learning is applied in studies of plant–pathogen interaction including disease monitoring, the discovery of gene regulatory networks and genomic selection for disease resistance [181]. The category of unsupervised machine learning is of special interest, whereas there is no specification about the outcome in the data set with clustering and “feature” extraction [182]. It is a new paradigm where traditional stress-related traits could be substituted by synthetic features possibly more related to multiple-stress conditions that could be important for investigating crop resilience under climate change.

## Figures and Tables

**Table 1:** Transgenic six-stacked maize varieties in commercial use *.

ISAAA Event	Code (Unique Identifier)	Trade Name	Herbicide Tolerance	Insect Resistance	Drought Tolerance	Modified Starch	Selectable Marker
3272 × Bt11 × 59,122 × MIR604 × TC1507 × GA21	SYN-E3272-5 × SYN-BTØ11-1 × DAS-59122-7 × SYN-IR6Ø4-5 × DAS-Ø15Ø7-1 × MON-ØØØ21-9	not available	1. glyphosate (*mepsps*) 2. glufosinate (*pat*)	1. Cry1Ab delta-endotoxin (*cry1Ab*) 2. Cry34Ab1 delta-endotoxin (*cry34Ab1*) 3. Cry35Ab1 delta-endotoxin (*cry35Ab1*) 4. modified Cry3A delta-endotoxin (*mcry3A*) 5. modified Cry1F protein (*cry1Fa2*)	none	1. thermostable α-amylase aa(*amy797E*)	1. phosphomannose aaisomerase (*pmi*)
3272 × Bt11 × MIR604 × TC1507 × 5307 × GA21	SYN-E3272-5 × SYN-BTØ11-1 × SYN-IR6Ø4-5 × DAS-Ø15Ø7-1 × SYN-Ø53Ø7-1 × MON-ØØØ21-9	not available	1. glyphosate (*mepsps*) 2. glufosinate (*pat*)	1. Cry1Ab delta-endotoxin (*cry1Ab*) 2. modified Cry3A delta-endotoxin (*mcry3A*) 3. modified Cry1F protein (*cry1Fa2*) 4. synthetic form of Cry3A and Cry1Ab (*ecry3.1Ab*)	none	1. thermostable α-amylase aa(*amy797E*)	1. phosphomannose ccisomerase (*pmi*)
5307 × MIR604 × Bt11 × TC1507 × GA21 × MIR162	SYN-Ø53Ø7-1 × SYN-IR6Ø4-5 × SYN-BTØ11-1 × DAS-Ø15Ø7-1 × MON-ØØØ21-9 × SYN-IR162-4	Agrisure^®^ Duracade™ 5222	1. glyphosate (*mepsps*) 2. glufosinate (*pat*)	1. Cry1Ab delta-endotoxin (*cry1Ab*) 2. modified Cry3A delta-endotoxin (*mcry3A*) 3. modified Cry1F protein (*cry1Fa2*) 4. chimeric Cry3A-Cry1Ab delta (*ecry3.1Ab*) 5. vegetative insecticidal protein (*vip3Aa20*)	none		1. phosphomannose aaisomerase (*pmi*)
Bt11 × MIR162 × MIR604 × MON89034 × 5307 × GA21	SYN-BTØ11-1 × SYN-IR162-4 × SYN-IR6Ø4-5 × MON-89Ø34-3 × SYN-Ø53Ø7-1 × MON-ØØØ21-9	not available	1. glyphosate (*mepsps*) 2. glufosinate (*pat*)	1. Cry1Ab delta-endotoxin (c*ry1Ab*) 2. modified Cry3A delta-endotoxin (*mcry3A*) 3. vegetative insecticidal protein (*vip3Aa20*) 4. Cry2Ab delta-endotoxin (c*ry2Ab2*) 5. chimeric Cry1Ab-Cry1F-Cry1Ac (*cry1A.105*) 6. chimeric Cry3A-Cry1Ab delta (*ecry3.1Ab*)	none		none
MON87427 × MON89034 × MON810 × MIR162 × MON87411 × MON87419	MON-87427-7 × MON-89Ø34-3 × MON-ØØ81Ø-6 × SYN-IR162-4 × MON-87411-9 × MON87419-8	not available	1. glyphosate (*cp4 epsps*) 2. glufosinate (*pat*) 3. dicamba (*dmo*)	1. Cry1Ab delta-endotoxin (*cry1Ab*) 2. vegetative insecticidal protein (*vip3Aa20*) 3. Cry2Ab delta-endotoxin (Cry2Ab2) 4. chimeric Cry1Ab-Cry1F-Cry1Ac (*cry1A.105*) 5. Cry3Bb1 delta endotoxin (*cry3Bb1*)	none		1. glyphosate oxidase aa(*gox247*) 2. neomycin phosphotransferase aa(*nptII*) 3. phosphomannose aaisomerase (*pmi*)
MON87427 × MON87460 × MON89034 × TC1507 × MON87411 × 59122	MON-87427-7 × MON-8746Ø-4 × MON-89Ø34-3 × DAS-Ø15Ø7-1 × MON-87411-9 × DAS-59122-7	not available	1. glyphosate (*cp4 epsps*) 2. glufosinate (*pat*)	1. Cry1F delta-endotoxin (*cry1F*) 2. Cry34Ab1 delta-endotoxin (*cry34Ab1*) 3. Cry3Bb1 delta endotoxin (*cry34Bb1*) 4. Cry2Ab delta-endotoxin (c*ry2Ab2*) 5. chimeric Cry1Ab-Cry1F-Cry1Ac (*cry1A.105*) 6. Cry3Bb1 delta endotoxin (*cry3Bb1*) 7. ds RNA (*dvsn7*)	1. cold shock protein B (*cspB*)		none
MON87427 × MON89034 × TC1507 × MON87411 × 59,122 × DAS40278	MON-87427-7 × MON-89Ø34-3 × DAS-Ø15Ø7-1 × MON-87411-9 × DAS-59122-7 × DAS-4Ø278-9	SmartStax™ Pro x Enlist™	1. glyphosate (*cp4 epsps*) 2. glufosinate (*pat*) 3. 2,4-D (*aad-1*)	1. Cry1F delta-endotoxin (*cry1F*) 2. Cry34Ab1 delta-endotoxin (*cry34Ab1*) 3. Cry35Ab1 delta endotoxin (*cry35Ab1*) 4. Cry2Ab delta-endotoxin (c*ry2Ab2*) 5. chimeric Cry1Ab-Cry1F-Cry1Ac (*cry1A.105*) 6. Cry3Bb1 delta endotoxin (*cry3Bb1*) 7. ds RNA (*dvsnf7*)	none		none
MON87427 × MON89034 × TC1507 × MON87411 × 59,122 × MON87419	MON-87427-7 × MON-89Ø34-3 × DAS-Ø15Ø7-1 × MON-87411-9 × DAS-59122-7 × MON87419-8	not available	1. glyphosate (*cp4 epsps*), 2 cassettes 2. glufosinate (*pat*), 3 cassettes 3. dicamba (*dmo*)	1. modified Cry1F protein (*cry1Fa2*) 2. Cry34Ab1 delta-endotoxin (*cry34Ab1*) 3. Cry35Ab1 delta endotoxin (*cry35Ab1*) 4. Cry2Ab delta-endotoxin (c*ry2Ab2*) 5. chimeric Cry1Ab-Cry1F-Cry1Ac (*cry1A.105*) 6. Cry3Bb1 delta endotoxin (*cry3Bb1*) 7. ds RNA (*dvsnf7*)	none		none

* Information was obtained from the ISSSA GM Approval Database: https://www.isaaa.org/gmapprovaldatabase/crop/default.asp? accessed on 9 September 2021. CropID=6&Crop=Maize. Code acronyms: MON—Monsanto, SYN—Syngenta, DAS—Dow AgroSciences.
